# Supplementary material for: Triapine Derivatives Act as Copper Delivery Vehicles to Induce Deadly Metal Overload in Cancer Cells
Source: Biomolecules. 2020 Sep 19;10(9):1336. doi: 10.3390/biom10091336 (PMC7564244; doi:10.3390/biom10091336)
Supplement: Supplementary file 1 [file biomolecules-10-01336-s001.pdf]

Supporting Information

for

Triapine Derivatives Act as Copper Delivery Vehicles to Induce Deadly Metal Overload in Cancer Cells

Kateryna Ohui,[a,#] Iryna Stepanenko,*[,a,#] Iuliana Besleaga,[a] Maria V. Babak,[b,c] Radu Stafi,[a] Denisa Darvasiova,[d] Gerald Giester,[e] Vivien Pósa,[f,g] Éva A. Enyedy,[f,g] Daniel Vegh,[h] Peter Rapta,[d] Wee Han Ang,[b,c] Ana Popović-Bijelić,[i] Vladimir B. Arion*[,a]

[a]*University of Vienna, Institute of Inorganic Chemistry, Währinger Strasse 42, A-1090 Vienna, Austria*

[b]*Department of Chemistry, National University of Singapore, 3 Science Drive 2, 117543 Singapore*

[c]*Drug Development Unit, National University of Singapore, 28 Medical Drive, 117546 Singapore*

[d]*Institute of Physical Chemistry and Chemical Physics, Slovak Technical University of Technology, Radlinského 9, 81237 Bratislava, Slovak Republic*

[e]*University of Vienna, Department of Mineralogy and Crystallography, Althan Strasse 14, A-1090 Vienna, Austria*

[f]*Department of Inorganic and Analytical Chemistry, Interdisciplinary Excellence Centre, University of Szeged, Dóm tér 7, H-6720 Szeged, Hungary*

[g]*MTA-SZTE Lendület Functional Metal Complexes Research Group, University of Szeged, Dóm tér 7, H-6720 Szeged, Hungary*

[h]*Institute of Organic Chemistry, Catalysis and Petrochemistry, Department of Organic Chemistry, Slovak Technical University of Technology, Radlinského 9, 81237 Bratislava, Slovak Republic*

[i]*Faculty of Physical Chemistry, University of Belgrade, 11158 Belgrade, Serbia*

[#] both co-authors contributed equally

* corresponding author: vladimir.arion@univie.ac.at; iryna.stepanenko@univie.ac.at

Content

Synthesis of **HL^{1a}** along with synthetic pathway (Scheme S1) S3

Tautomeric forms of **HL^{1}** and **HL^{2}** (Scheme S2) S4

Atom numbering used for assignments in 1D and 2D NMR spectra of **HL^{1}** and **HL^{2}** (Scheme S3) S4

The tautomeric forms of **HL^{2}** in solution (Scheme S4) S4

Preparative HPLC trace of $[Cu(HL^1)Cl_2]$ (**1**) (Figure S1) S5

ESI mass spectra of **1**$\cdot H_2O$ after HPLC (Figure S2) S5

Positive ion ESI mass spectrum of **3**$\cdot 0.75H_2O$ (Figure S3) S6

The structure of the cation $[\mathbf{H_3L^{1a}}]^{2+}$ in $[\mathbf{H_3L^{1a}}]Cl_2\cdot 2H_2O$ (Figure S4) S6

pH-potentiometric titration curves for **HL^{2}** (Figure S5) S7

UV-Vis spectra of **HL^{1}** recorded at various pH values (Figure S6) S7

Time dependence of the measured spectra of **HL^{2}** at various pH values (Figure S7) S8

Time dependence of the measured spectra of **HL^{1}** at various pH values (Figure S8) S9

Time-dependent UV–Vis spectra of (a) **HL^{1}** and (b) **HL^{2}** at pH 2.6 (Figure S9) S9

Time-dependent UV–Vis spectra of (a) **1** and (b) **2** at various pH values (Figure S10) S10

Concentration-effect curves for **HL^{1}**, **HL^{2}** and **1**–**3** (Figure S11) S10

UV–Vis spectra of (a) **1** and (b) **2** in the presence of 120 equiv GSH (Figure S12) S11

EPR spectra of DMPO spin-adducts (Figures S13 and S14) S11, S12

Synthesis of HL^{1a}: To 3-*N*-(*tert*-butyloxycarbonyl)amino-2-pyridinecarboxaldehyde (210 mg, 0.945 mmol) and 2-pyridinamidrazone (128.65 mg, 0.945 mmol) in ethanol/water (3/1, 8 mL) 12M HCl (0.173 mL, 2.08 mmol) was added dropwise. The resulting solution was stirred at room temperature for 2 h, then additional amount of 12M HCl (0.173 mL, 2.08 mmol) was added and the reaction mixture heated overnight at 85 °C to produce a yellow suspension. The yellow precipitate of [$\mathbf{H_3L^{1a}}$]$Cl_2 \cdot 2.5H_2O$ was collected by filtration and dried in vacuo. The raw product was neutralised with saturated $NaHCO_3$ until pH 7-8 to produce $\mathbf{HL^{1a}}$ ($C_{12}H_{12}N_6$, 70 mg, 0.29 mmol, yield 31%). From the first filtrate (before neutralization) the yellow needle-like crystalline product of [$\mathbf{H_3L^{1a}}$]$Cl_2 \cdot 2H_2O$ of X-ray diffraction quality was isolated. Anal. Calcd for [$\mathbf{H_3L^{1a}}$]Cl_2 $2.5H_2O$ (*Mr* = 358.22), %: C, 40.23; H, 5.35; N, 23.46. Found, %: C, 40.17; H, 5.26; N, 23.32. Anal. Calcd for $\mathbf{HL^{1a}} \cdot 0.1H_2O$ (M_r = 242.07), %: C, 59.54; H, 5.08; N, 34.72. Found, %: C, 59.63; H, 4.94; N, 34.39. ^{1}H NMR ($\mathbf{HL^{1a}}$, 500 MHz, DMSO-d_6), δ, ppm: 8.86 (s, 1H), 7.95 (d, *J* = 2.9 Hz, 1H), 7.43 – 6.95 (m, 4H). ESI-MS for $\mathbf{HL^{1a}}$ (MeCN/MeOH+1% H_2O) positive: *m/z* 241.23 [$\mathbf{HL^{1a}}$+H]$^+$, negative: *m/z* 239.11 [$\mathbf{HL^{1a}}$–H]$^-$.

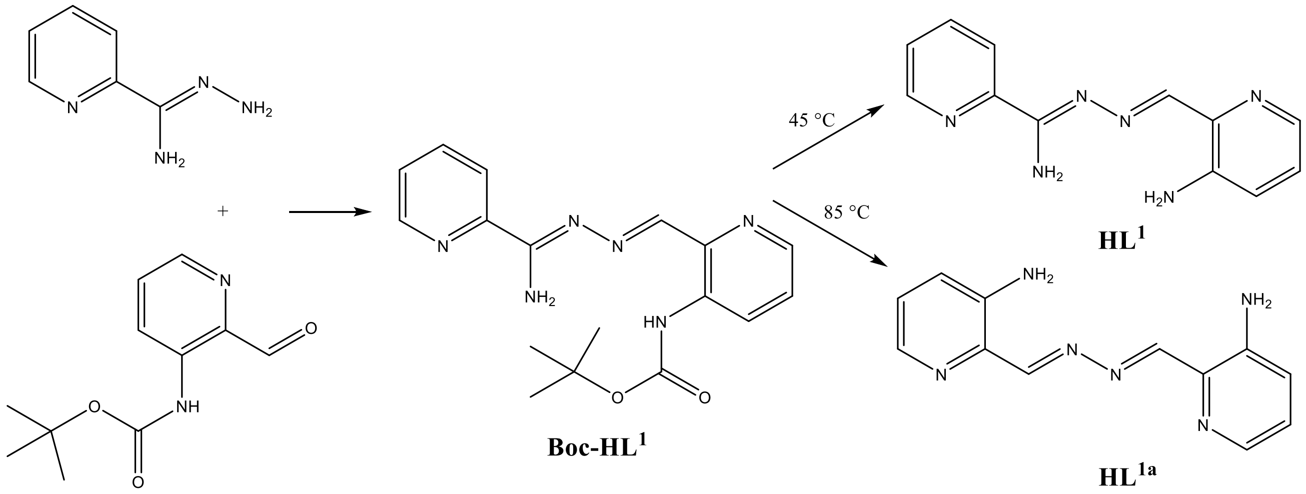

Scheme S1. Synthesis of $\mathbf{HL^1}$ and $\mathbf{HL^{1a}}$.

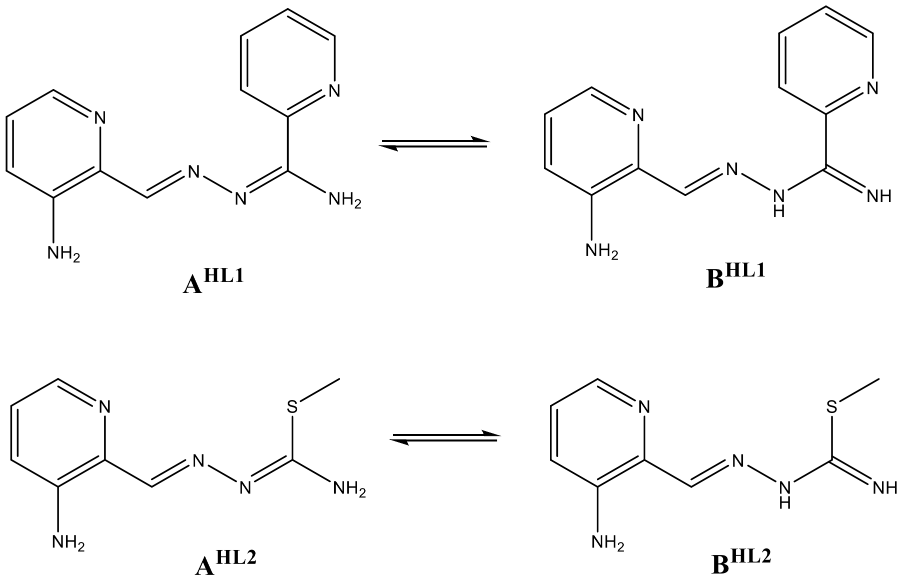

Scheme S2. The likely tautomeric forms for $\mathbf{HL^1}$ and $\mathbf{HL^2}$.

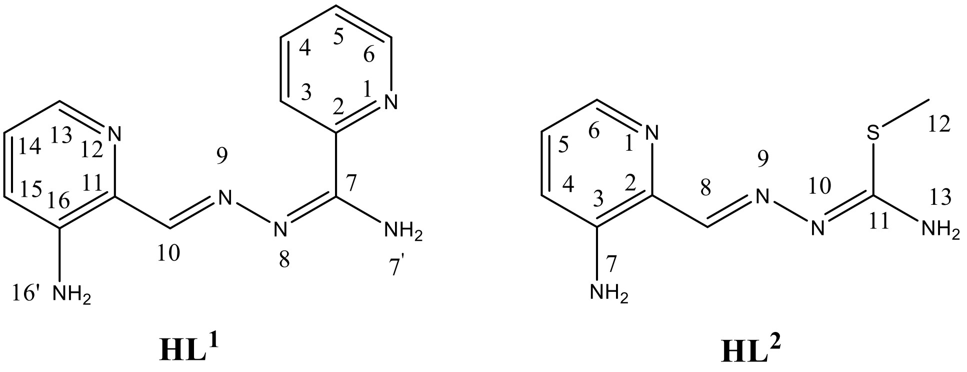

Scheme S3. Atom numbering used for assignments in 1D and 2D NMR spectra of $\mathbf{HL^1}$ and $\mathbf{HL^2}$.

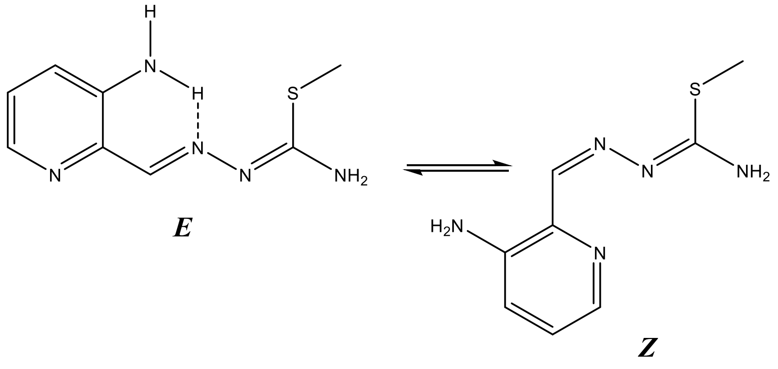

Scheme S4. The tautomeric forms of $\mathbf{HL^2}$ in solution.

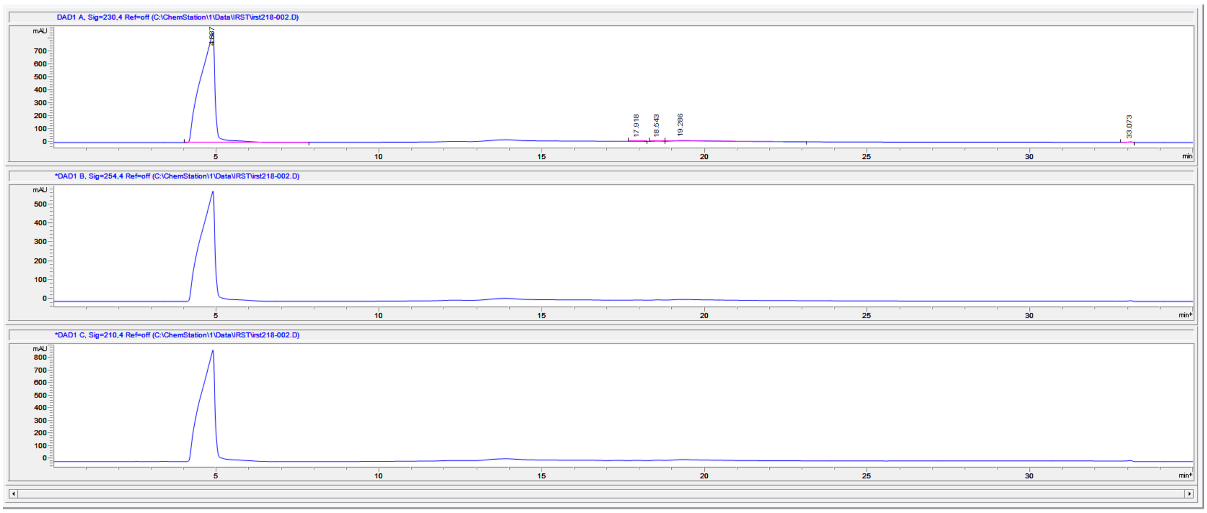

Figure S1. Preparative HPLC trace of [Cu(HL[1])Cl_2] (**1**).

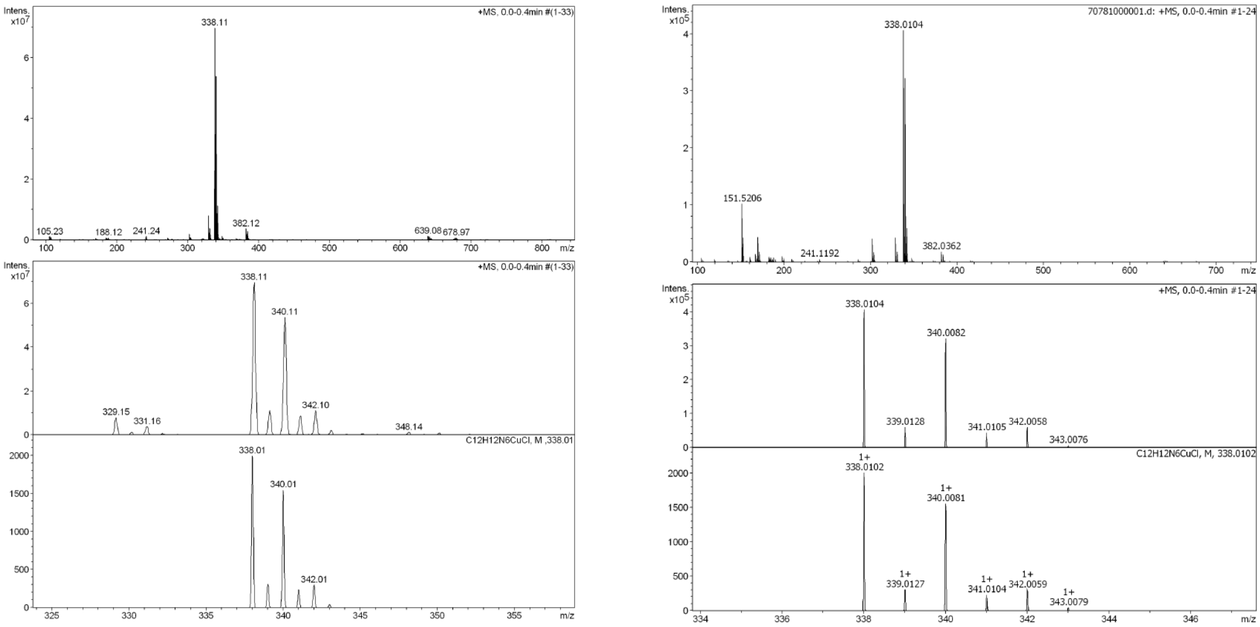

ESI MS

High resolution ESI MS

Figure S2. ESI mass spectra of **1**·H_2O after HPLC.

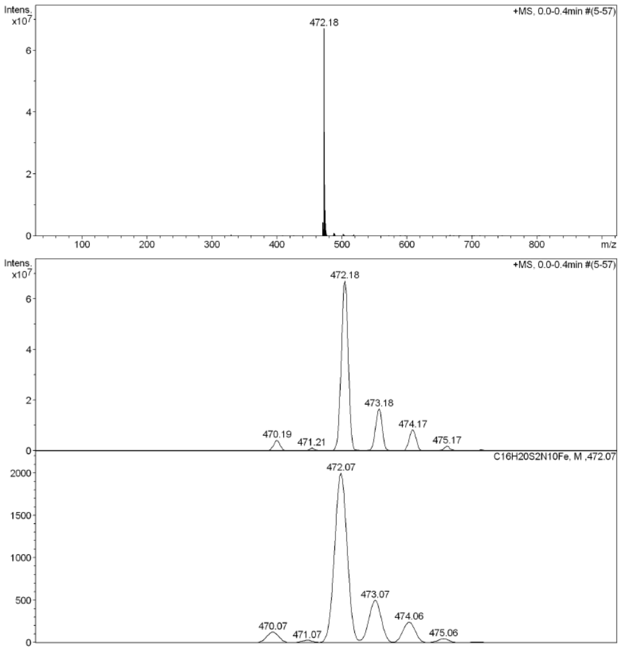

Figure S3. Positive ion ESI mass spectrum of **3**$\cdot 0.75H_2O$. The peak at *m*/*z* 472.18 is attributed to $[Fe(L^2)_2]^+$.

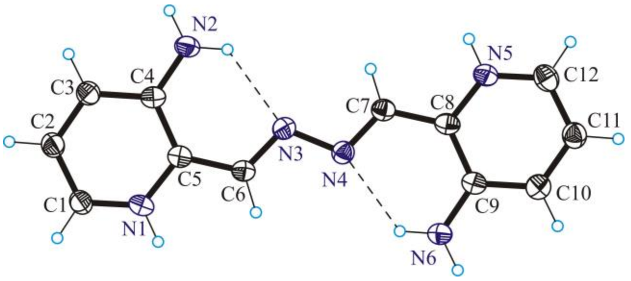

Figure S4. The structure of the cation $[\mathbf{H_3L^{1a}}]^{2+}$ in $[\mathbf{H_3L^{1a}}]Cl_2\cdot 2H_2O$. Selected bond distances (Å) and torsion angles (deg): C4–N2 1.339(4), C4–C5 1.422(5), C5–C6 1.431(5), C6–N3 1.291(4), N3–N4 1.398(4), N4–C7 1.290(4), C7–C8 1.437(5), C8–C9 1.421(5), C9–N6 1.342(4); C6–N3–N4–C7 –179.3(3). H-bond parameters: N2–H···N3 [N2···N3 2.778(4) Å, N2–H···N3 129.4°]; N6–H···N4 [N6···N4 2.772(4) Å, N6–H···N4 129.7°].

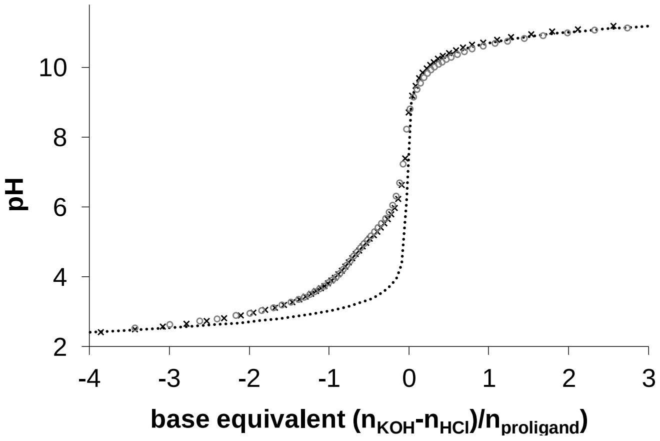

Figure S5. pH-potentiometric titration curves for **HL[2]:** direct (×) and back-titration (o); and for HCl (dotted line) for comparison in pure water. {*c_L = 3 mM; T = 298 K; I = 0.10 M (KCl)*}

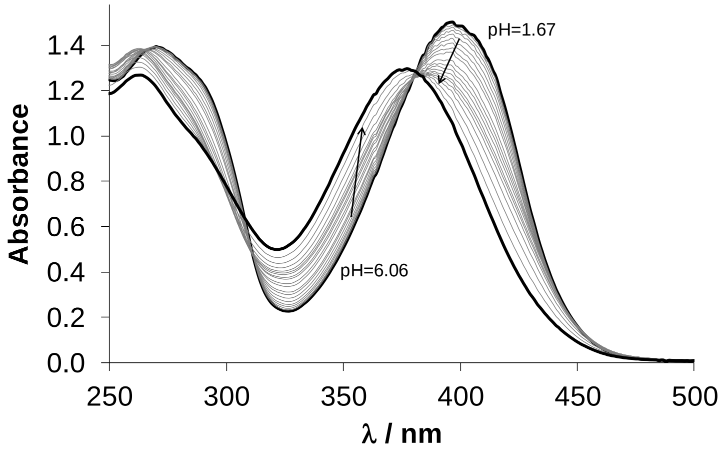

Figure S6. UV–Vis spectra of **HL[1]** recorded at various pH values. {*c_L = 100 μM; T = 298 K; I = 0.10 M (KCl); ℓ = 1.0 cm; 1% (v/v) DMSO*}

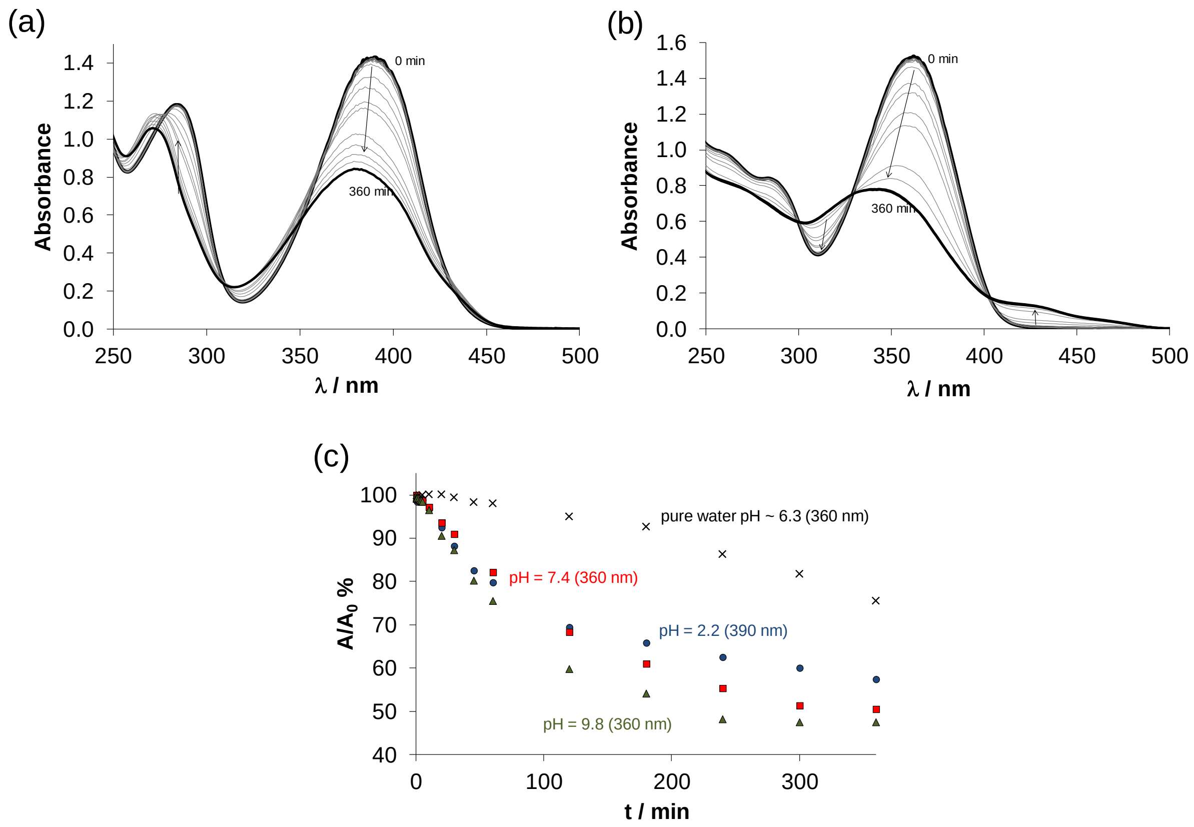

Figure S7. Time dependence of the measured spectra of **HL**^{2} at various pH values: (a) 2.2; (b) 9.8 and changes of the absorbance values (as A/A_0) at the different pH values and in pure water (without buffer and KCl, pH = 6.3) {*c_L = 100 μM; T = 298 K; I = 0.10* M *(KCl); ℓ = 1.0 cm; 1% (v/v) DMSO*}.

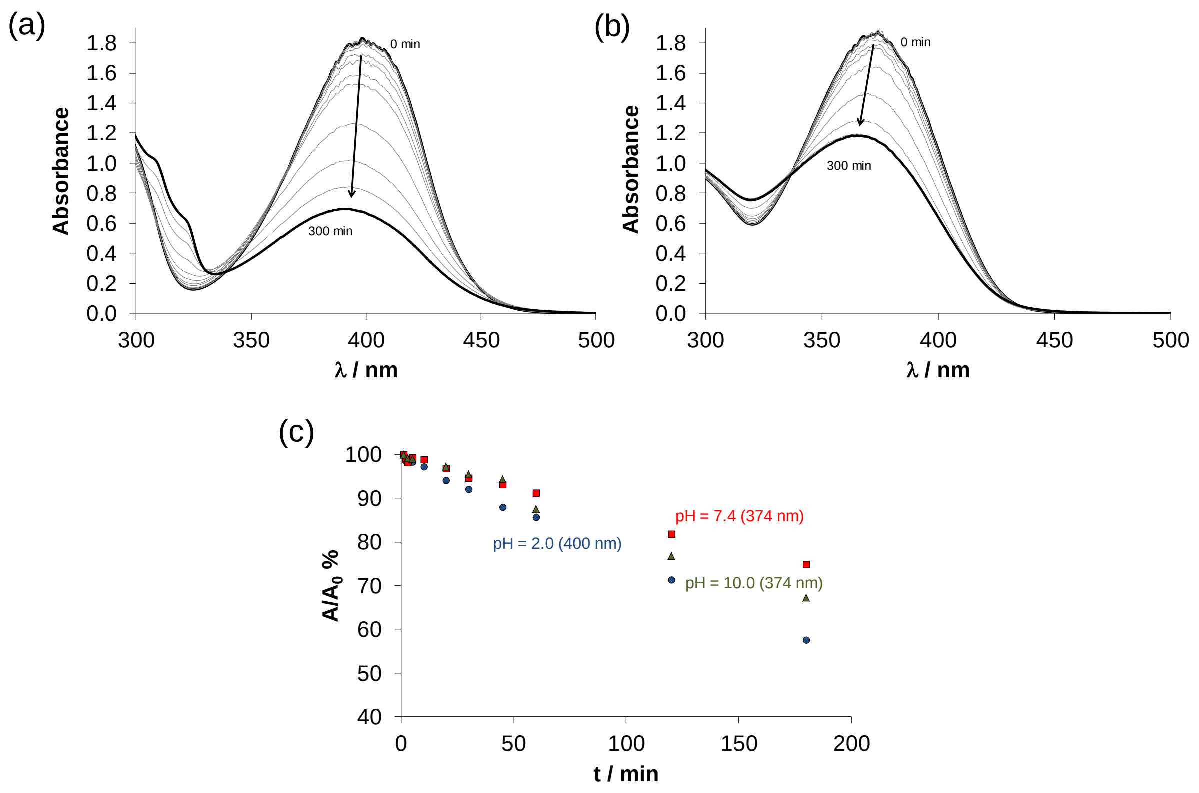

Figure S8. Time dependence of the measured spectra of **HL[1]** at various pH values: (a) 2.0; (b) 10.0 and (c) changes of the absorbance values (as A/A_0) {*c_L = 100 μM; T = 298 K; I = 0.10* M *(KCl); ℓ = 1.0 cm; 1% (v/v) DMSO*}.

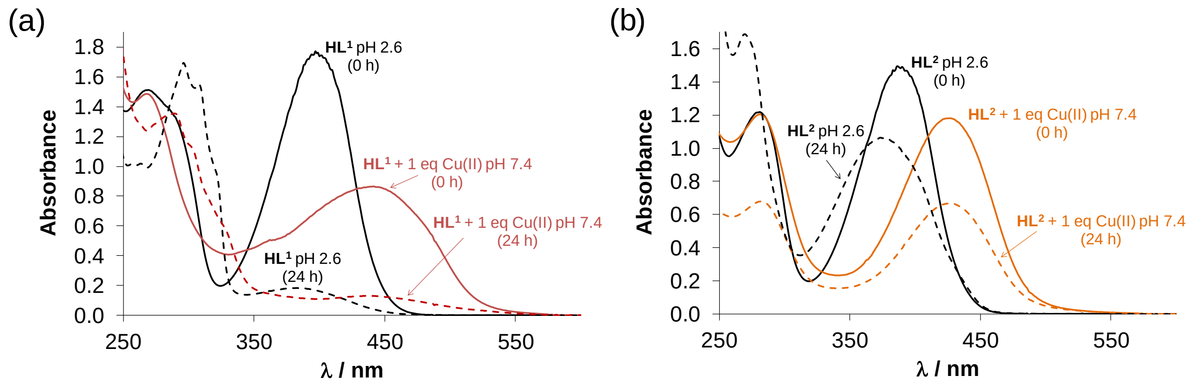

Figure S9. UV–Vis spectra of (a) **HL[1]** and (b) **HL[2]** at pH 2.6 measured immediately (0 h) and after 24 h in addition to spectra recorded for the ligand samples kept for 0 or 24 h at pH 2.6 in the presence of 1 equiv Cu(II) ion at pH 7.4 {*c_L = 100 μM; T = 298 K; I = 0.10* M *(KCl); ℓ = 1.0 cm; 1% (v/v) DMSO*}.

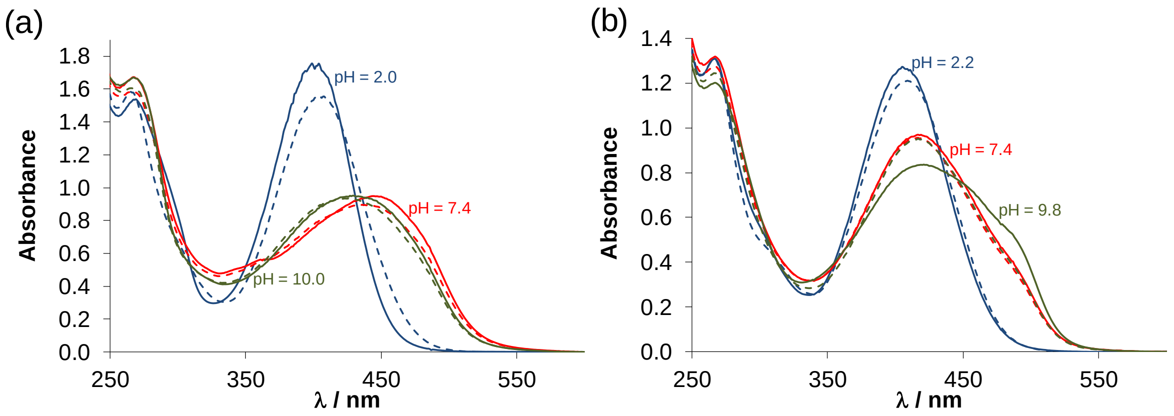

Figure S10. UV–Vis spectra of (a) **1** and (b) **2** at various pH values measured immediately upon dissolution (solid lines) and after 24 h (dashed lines) {*$c_{complex}$ = 100 µM; T = 298 K; I = 0.10 M (KCl); ℓ = 1.0 cm; 1% (v/v) DMSO*}.

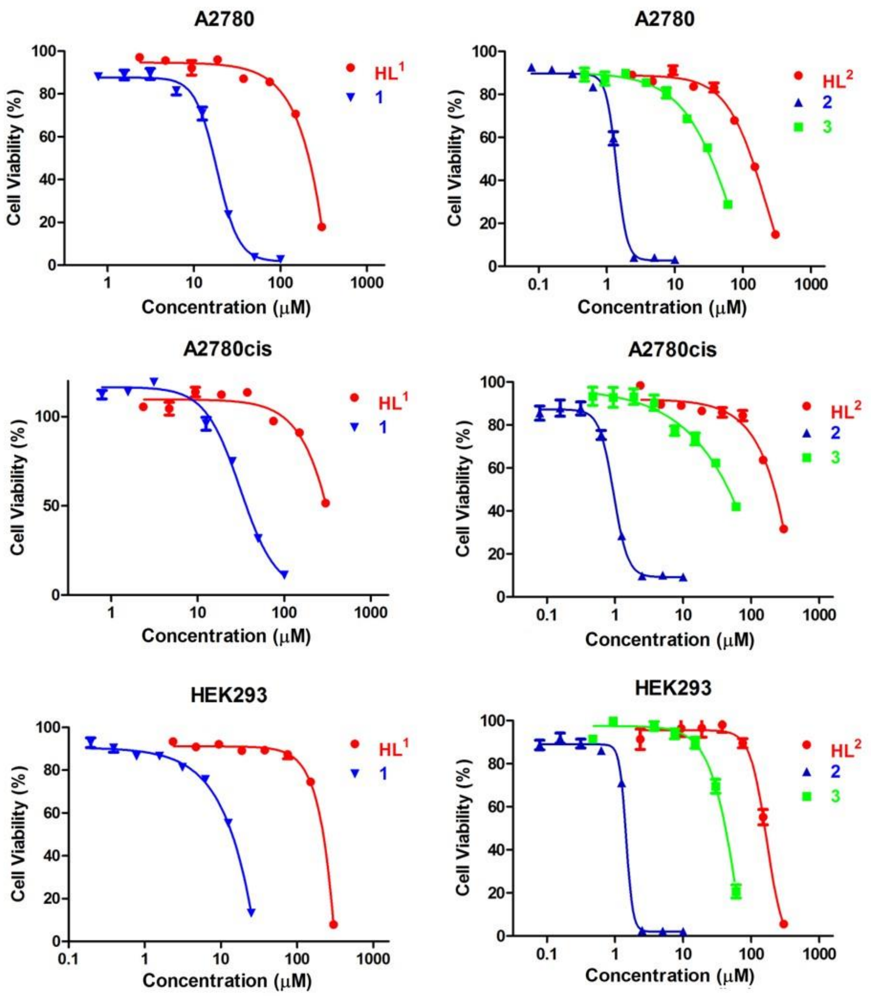

Figure S11. Concentration-effect curves for **HL¹**, **HL²** and **1**–**3** in A2780, A2780cis and HEK293 cell lines upon 72 h exposure.

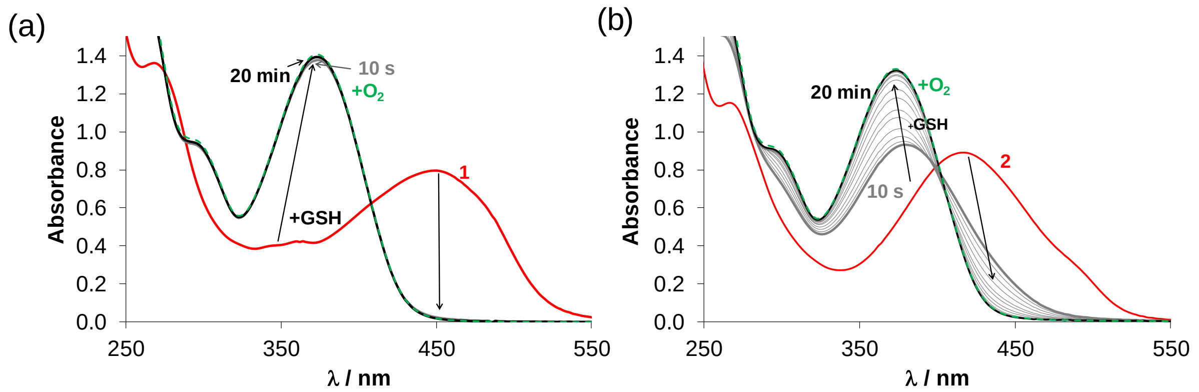

Figure S12. UV–Vis spectra of (a) **1** and (b) **2** in the presence of 120 equiv GSH before (red lines) and after (black and grey lines) mixing their solutions in a tandem cuvette, and the effect of the addition of O_2 to the sample followed by the reaction (green dashed lines) {$c_{complex}$ = *100 μM*; c_{GSH} = *12.0 mM; pH = 7.40 (50 mM HEPES); T = 298 K; I = 0.10 M (KCl); ℓ = 1.0 cm; 1% (v/v) DMSO*}.

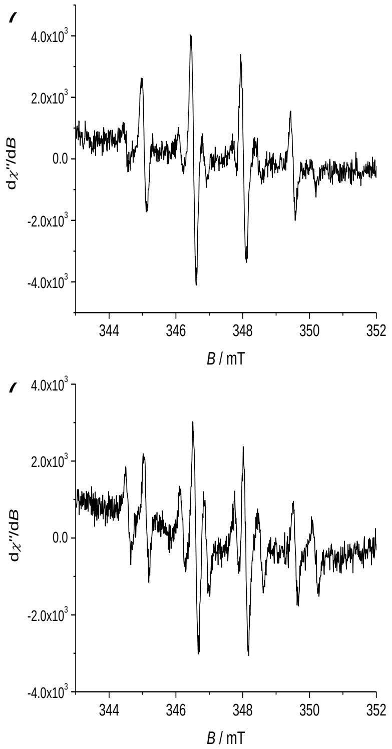

Figure S13. EPR spectra of DMPO spin-adducts measured in the 1% (v/v) DMSO/H_2O) + DMPO + (a) **Fe(II)-(HL^2)$_2$** or (b) **Fe(II)-(3AP)$_2$** (prepared by the reaction of the corresponding ligand with $FeSO_4 \cdot 6H_2O$ at 1:2 iron-to-ligand mole ratio) + H_2O_2 system measured on air after 2 min of reactions: Initial concentrations: c_0(**Fe(II)-(HL^2)$_2$**) or c_0**Fe(II)-(3AP)$_2$** = 0.4 mM, c_0(H_2O_2) = 0.01 M, c_0(DMPO) = 0.04 M.

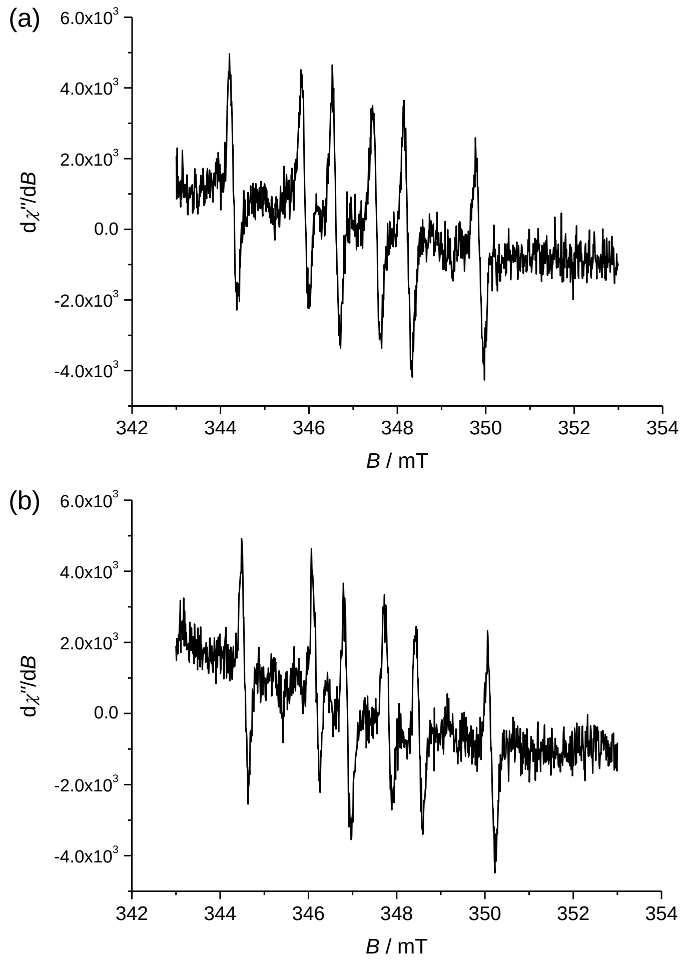

Figure S14. EPR spectra of DMPO spin-adducts measured in 5% (v/v) DMSO/H_2O) + DMPO + (a) **Fe(II)-(HL^2)$_2$** or (b) **Fe(II)-(3AP)$_2$** (prepared by the reaction of the corresponding ligand with $FeSO_4 \cdot 6H_2O$ at 1:2 iron-to-ligand mole ratio) + H_2O_2 system measured on air after 10 min of reactions: Initial concentrations: c_0(**Fe(II)-(HL^2)$_2$**) or c_0**Fe(II)-(3AP)$_2$** = 0.4 mM, c_0(H_2O_2) = 0.01 M, c_0(DMPO) = 0.04 M.
